# Temporal Associations between T-Cell Senescence and Arterial Stiffness in Middle-Aged /Older Adults

**DOI:** 10.1093/geroni/igaf122.3663

**Published:** 2025-12-31

**Authors:** Theodore DeConne, Stephen Kritchevsky, Jamie Justice, Jingzhong Ding

**Affiliations:** Wake Forest University School of Medicine, Winston-Salem, North Carolina, United States; Wake Forest University School of Medicine, Winston-Salem, North Carolina, United States; Wake Forest University School of Medicine, Winston-Salem, North Carolina, United States; Wake Forest University School of Medicine, Winston-Salem, North Carolina, United States

## Abstract

Age-related structural arterial remodeling (S) and load-dependent (LD) arterial stiffening can be calculated using arterial pulse-wave velocity (PWV) and blood pressure. Senescence-associated T-cells are cross-sectionally associated with both S-PWV and LD-PWV; however, the temporality isn’t known. This analysis aimed to determine whether higher T-cell senescence precedes or follows higher S-PWV and LD-PWV leveraging data from the VEGGIE trial. The VEGGIE trial was an 18-week randomized, controlled, caloric restriction weight-loss trial in middle-aged and older adults (age:57.6 ± 5.8 years, n = 58, systolic blood pressure=129±17mmHg at baseline). PWV was estimated (ePWV) using mean arterial pressure and age at each timepoint with previously established reference values. S-ePWV and LD-ePWV were calculated from total ePWV (T-ePWV) using participant-specific exponential models. CD3+ T-cell senescence gene expression (p16INK4a, p21CIP1/WAF1, BCL2, and BAK1) was measured. Multivariable linear regressions tested associations between baseline T-cell senescence and arterial stiffness with 18-week change in PWV or T-cell senescence. Models were adjusted for biologic sex, BMI, race, and treatment-group. Higher baseline p16 (β = 0.546,P=0.0688) and p21 (β = 0.398,P=0.0593) were nominally associated with increased T-ePWV. Higher baseline BAK1 was associated with increased S-ePWV (β = 0.0987,P=0.037). A treatment-group-by-baseline p16 interaction effect was observed with delta T-ePWV (p = 0.0234), where T-ePWV only increased in participants with high p16 at baseline in the caloric restriction group (β = 1.16,P=0.00405). Interestingly, higher baseline T-ePWV (β=-0.329,P=0.0556) and S-ePWV (β=-0.378,P=0.048) were associated with decreased p21. In conclusion, temporal associations between CD3+ senescence and arterial stiffness may be bi-directional and future research is needed directly measuring PWV and T-cell senescence over longer periods.

